# Prevalence and Predictors of Work–Life Balance Among Nursing Personnel During the Sixth Wave of the Pandemic: The Role of Stress and Sociodemographic and Work-Related Variables

**DOI:** 10.3390/bs15060751

**Published:** 2025-05-30

**Authors:** Ana María Antolí-Jover, María Gázquez-López, Pascual Brieba-del Río, Adelina Martín-Salvador, Encarnación Martínez-García, Inmaculada Sánchez-García, María Adelaida Álvarez-Serrano

**Affiliations:** 1 Department of Nursing, Faculty of Health Sciences, University of Granada, 51001 Ceuta, Spain; antolijover@ugr.es (A.M.A.-J.); adealvarez@ugr.es (M.A.Á.-S.); 2 Instituto Nacional de Gestión Sanitaria, 51002 Ceuta, Spain; pbrieba@ingesa.sanidad.gob.es; 3 Department of Nursing, Faculty of Health Sciences, University of Granada, 18016 Granada, Spain; ademartin@ugr.es; 4 Virgen de las Nieves, University Hospital, 18014 Granada, Spain; 5 Department of Nursing, Faculty of Health Sciences, University of Jaén, 23071 Jaén, Spain; isgarcia@ujaen.es

**Keywords:** work–life balance, work–life conflict, family support, occupational stress, nursing, health personnel, COVID-19

## Abstract

The COVID-19 pandemic has intensified the challenges of balancing work and personal life for healthcare professionals, particularly nurses. In Spain, during the sixth wave of the pandemic, characterized by a high number of infections and increased healthcare pressure, these challenges became even more pronounced. This study examines how perceived stress, sociodemographic characteristics, and work-related factors influence Work–Life Balance among Spanish nurses in this context. A cross-sectional study was conducted with a sample of 305 Spanish nurses using the online Work-Life Interaction Questionnaire (SWING) and the Perceived Stress Scale (EP-10). The findings revealed that higher levels of perceived stress were associated with increased work-to-family conflict. This conflict was further intensified among nurses working rotating shifts. In the reverse direction, from life to work, perceived stress was again a significant factor, and having children contributed to increased negative life-to-work interference. On the other hand, certain variables were linked to more positive interactions. Having a paid caregiver was associated with lower positive work-to-family interaction, whereas religious beliefs were related to more positive experiences in this domain. Finally, being a woman and having children were both associated with greater positive life-to-work interaction. This study offers a vital perspective on the complex Work–Life interaction in nursing during crises, highlighting the urgent need for structural policies that alleviate stress and conflict while enhancing well-being by recognizing the protective role of family and spirituality. These findings open new avenues for designing more effective, responsive interventions for healthcare personnel.

## 1. Introduction

In recent decades, interest in Work–Life Balance, understood as the ability to effectively manage and harmonize work and family responsibilities, has grown significantly, motivated by growing evidence of the negative effects that arise when the two domains are not adequately integrated (Grzywacz & Marks, 2000; Livingston & Judge, 2008). Until now, research has mainly focused on Work–Life conflict, exploring how the demands of one domain can interfere with the performance of the other. In this regard, Greenhaus and Beutell (1985) conceptualized ‘Work–Life conflict’ as a form of role conflict, in which the demands of work and family are, in many cases, incompatible (Greenhaus & Beutell, 1985). Based on this definition, studies have examined the two directions of the conflict: how work responsibilities affect family responsibilities (Work–Life conflict) and how family demands interfere with work performance (Work–Life conflict) (Frone et al., 1992; Lu et al., 2020).

In recent years, a new research perspective has emerged, which seeks to analyze not only negative Work–Life interferences, but also possible positive interactions. Concepts such as ‘Work–Life facilitation’ and ‘Work–Life enrichment’ have gained prominence. Work–Life enrichment, defined as the degree to which experiences in one role improve the quality of life in other roles (Greenhaus et al., 2003), highlights that not all interactions between these domains are conflicting (Geurts et al., 2005). To comprehensively measure this interaction, instruments such as the SWING (Survey Work-Life Interaction-Nijmegen) by Geurts et al. (2005), which measures both positive and negative effects of Work–Life interaction, have been developed.

The extension of this approach has been important for a deeper understanding of how work experience can influence personal responsibilities and vice versa, highlighting that it is not always a conflict, but that there can be mutual facilitation between the two domains. This issue is of particular importance in health professions, such as nursing, where Work–Life Balance has direct implications for both the well-being of professionals and the quality of care received by patients. An appropriate Work–Life Balance allows nurses to perform their work more effectively, with greater empathy and communication with patients and their families, which in turn contributes to improved clinical outcomes and patient satisfaction (Bodendieck et al., 2022). In this sense, further understanding of the factors that influence this balance becomes a priority, as a better understanding of these factors can guide the implementation of workplace policies that promote a healthier environment and reduce turnover in the healthcare workforce (de Vries et al., 2023).

One of the most decisive factors in the imbalance between nurses’ professional and personal lives is shift work. This organizational modality—especially in its rotating and night shift forms—is a constant feature of the hospital setting and represents a significant source of occupational stress (Emmanuel et al., 2024). Prolonged exposure to such schedules severely disrupts circadian rhythms, compromises restorative sleep, and fosters chronic fatigue. Added to this is the difficulty of maintaining family and social bonds, which are fundamental pillars of emotional well-being. This sustained disconnection from personal life can lead to emotional exhaustion and a sense of detachment—key components of burnout syndrome. Burnout, defined as a state of physical, emotional, and mental exhaustion caused by prolonged work-related stress (Maslach & Leiter, 2016), can reduce professional motivation and negatively affect the quality of care provided (Williams et al., 2022).

The COVID-19 pandemic not only brought these pre-existing tensions to light but critically intensified them—particularly for nurses working in high-demand units such as intensive care and emergency services (Domínguez-Salas et al., 2020). Increased workload, longer shifts, and constant exposure to biological risk heightened the physical and emotional strain, making it even more difficult to fulfill family and social roles. This prolonged imbalance weakened coping mechanisms and reduced access to essential support networks, becoming a key driver of chronic stress and burnout. In this context, it is imperative to implement effective emotional support and psychological care strategies to safeguard the mental health of professionals working under extreme conditions (de Vries et al., 2023).

During the sixth wave of COVID-19 infections in Spain (November 2021 to February 2022), nurses faced an exceptionally intense and prolonged period of professional and personal strain. This was due to two key factors. First, healthcare professionals had already endured nearly two years of relentless pressure since the start of the pandemic, leading to accumulated physical and emotional exhaustion. Second, the Omicron variant, although generally less severe than previous strains, was highly transmissible. This caused a sharp increase in cases, resulting in hospital overcrowding and further straining already depleted healthcare teams (Serrano-Cumplido et al., 2024).

Although numerous studies documented increased levels of anxiety, depression, and stress among nurses during the earlier waves, there is still a significant gap in the literature regarding how these stressors impacted their HRQoL and their capacity to reconcile professional and family responsibilities during the later stages of the crisis. Most existing research has examined either the professional quality of life or psychological symptoms in isolation (Luceño-Moreno et al., 2022; Sánchez-Sánchez et al., 2021), without incorporating broader and interconnected dimensions such as the HRQoL and Work–Life Balance. This lack of an integrated perspective limits our understanding of the long-term consequences of the pandemic on the nursing workforce. 

The relevance of this gap becomes even more apparent in light of recent national data from the General Council of Nursing’s 2023 macro-survey. The report highlights widespread professional dissatisfaction: over 8000 nurses left their jobs, citing job insecurity, seasonal contracts, and poor working conditions (Linde, 2024). Moreover, 60% of the nurses surveyed had considered leaving the profession and more than 30% would not choose nursing again. These figures reflect a profession under severe strain and emphasize the urgent need for institutional measures that promote healthier, more sustainable working conditions, particularly for those most vulnerable to the effects of Work–Life conflict.

Also, sociodemographic characteristics, such as age, marital status, the presence of dependent children, and years of service, are important factors in identifying nurses who are more vulnerable to Work–Life conflict. Studies suggest that nurses with less experience tend to have fewer tools to manage work-related stress, while those with more years of service are more exposed to chronic fatigue (Serrano-Ripoll et al., 2020; van der Heijden et al., 2010; Velásquez & Tovar, 2017). In addition, those with additional family responsibilities, such as caring for young children or dependent family members, often face greater difficulties in balancing their work and personal lives, highlighting the importance of designing specific support measures for these groups (Schieman et al., 2021).

In this context, understanding the factors that influence nurses’ ability to reconcile their professional and personal lives during critical periods, such as the sixth wave of the COVID-19 pandemic, is essential not only for preserving their well-being but also for ensuring the sustainability of healthcare systems. Therefore, the present study aims to analyze the impact of stress, sociodemographic characteristics, and occupational variables on Work–Life Balance among nursing staff in Spain during this period.

## 2. Materials and Methods

### 2.1. Study Design and Population

This cross-sectional study collected data through an online survey conducted between November and December 2021. The inclusion criteria required participants to be registered nurses working at the time of the survey in either public or private hospitals or out-of-hospital healthcare centers across Spain. Nurses who were retired, on extended leave, or exclusively occupying administrative, non-clinical roles were excluded from participation.

The sample size was determined based on the total number of registered nurses in Spain reported by the National Institute of Statistics (INE) in 2020, amounting to 330,745 professionals. Using a confidence level of 95% and a 6% margin of error, the minimum sample size was calculated to be 267 respondents.

To reach a wide and representative sample, collaboration was sought from the 52 official nursing associations and the national and provincial delegations of the SATSE trade union, requesting their active collaboration. Through these entities, the invitation link to participate in the survey was distributed both in mainland Spain and in non-mainland territories. A snowball sampling strategy was used, which made it possible to obtain a diverse and representative sample of nurses, taking advantage of existing professional and trade union networks to encourage participation.

### 2.2. Procedures and Measures

Data were obtained via a self-administered questionnaire, requiring approximately 20 min for completion. The instrument comprised sections collecting detailed information on occupational, sociodemographic, cultural, and family-related variables, in addition to COVID-19-related clinical and exposure factors.

The second section included two standardized questionnaires: the Perceived Stress Questionnaire PSS-10 and the Work-Life Interaction Questionnaire (SWING).

Stress was assessed with the 10-item Perceived Stress Scale (PSS-10) (S. Cohen et al., 1983). This instrument is composed of 10 questions, of which 4 (items 4, 5, 7 and 8) assess a positive attitude towards a situation, while the remaining 6 reflect a negative attitude. Each item is answered on a five-choice Likert scale. The score obtained on the PSS-10 is interpreted according to the following cut-off points: a score of 13 or lower indicates low stress, between 14 and 26 corresponds to a moderate level, and 27 or higher reflects a high level of stress. The cut-off points used in the PSS-10 were similar to those observed in other COVID-19 studies (Campo-Arias et al., 2020; Pedrozo-Pupo et al., 2020).

The Work-Life Interaction Questionnaire (SWING), validated in Spanish samples, demonstrates high internal consistency, with Cronbach’s alpha coefficients ranging from 0.77 to 0.89 (Moreno & Sanz, 2009). It consists of 22 items, 8 of which measure negative Work–Life interaction, 4 measure negative Life–Work interaction, and 5 items each measure positive Work–Life and Life–Work interactions. A 4-point Likert scale (0 to 3) is used. Scores are interpreted as low interaction (0 to less than 1), medium interaction (1 to less than 2), and high interaction (2 to 3).

### 2.3. Ethical Issues

This study complies with the regulations of good clinical practice, contained in the European Directive 2001/20/EC and Law 14/2007, of 3 July, on biomedical research. The processing of personal data in health research is governed by Organic Law 3/2018, of 5 December, on Data Protection and Guarantee of Digital Rights. The protocol obtained a favorable resolution from the Biomedical Research Ethics Committee of the province of Granada (code SICEIA-2024-000278). It was authorized by the Dean of the Faculty of Health Sciences in Ceuta on 13 September 2021. The participants were made aware of the study’s goals and provided their consent to participate by selecting a designated checkbox.

### 2.4. Statistical Analysis

Descriptive statistics were computed for all variables, with categorical variables summarized as frequencies and percentages and continuous variables reported as means and standard deviations. The Kolmogorov–Smirnov test was applied to assess data normality, confirming the appropriateness of the parametric analyses.

Independent samples *t*-tests were conducted to evaluate group differences, with effect sizes estimated using Cohen’s d. Pearson’s correlation coefficients were calculated to examine associations between continuous variables. To identify potential predictors for each dimension of the Work-Life Interaction Questionnaire (SWING), four multiple linear regression models were fitted.

Statistical significance was set at *p* < 0.05 for all analyses. Data processing and analyses were performed using IBM SPSS Statistics for Macintosh, version 25.0 (IBM Corp., Armonk, NY, USA).

## 3. Results

The sample was mainly composed of women (*n* = 265; 86.8%) and people living in the Iberian Peninsula (*n* = 232; 76.1%) with a mean age of 38.8 years (SD = 11.383). The majority of the participants identified themselves as atheist or agnostic (*n* = 175; 57.4%). In terms of family factors, 74.7% (*n* = 228) lived with a partner and more than half had children (*n* = 175; 57.4%), with an average of 1.03 children (SD = 1.062). Finally, the vast majority did not delegate the care of their children to a paid caregiver (*n* = 270; 88.5%), while only a small percentage did so (*n* = 35; 11.5%) (Table 1).

Table 2 describes the salient job characteristics of the participants and their experience with COVID-19. The majority of the participants had less than 5 years of experience, 55.1% (*n* = 168). For the classification of the services, high or low exposure to patients with COVID-19 was considered. In this context, 66.6% (*n* = 203) worked in low-exposure areas, while 33.4% (*n* = 102) worked in high-exposure services, such as emergencies, COVID-19-specific units, or intensive care.

Regarding working hours, 85.9% of the participants worked full-time (*n* = 262). Regarding shift work, 59.7% of the respondents worked rotating shifts (*n* = 182) and 52.8% worked in shifts that included night shifts (*n* = 161), either on a fixed shift basis or combined with other day shifts. Only 16.1% were in management positions (*n* = 49).

Finally, regarding their experience with COVID-19, only 17.7% (*n* = 54) of the participants had been diagnosed with the disease, while 33.8% (*n* = 103) had had to isolate themselves due to close contact with infected people.

Regarding the dimensions of the SWING questionnaire, the results from the SWING questionnaire, as presented in Table 3, show the range of scores across its four dimensions. The dimension with the lowest average score was negative Work–Life Balance, with a mean of 0.46 (SD = 0.443). In contrast, the dimension reflecting positive Life–Work Balance exhibited the highest average score, with a mean of 1.95 (SD = 0.693). 

In terms of the perceived stress, the sample achieved an average score of 23.2 (SD = 3.24). The majority of the participants (76.7%) reported medium stress levels (Table 3).

Table 4 shows the negative and positive interactions between Work–Life and Life–Work according to sociodemographic variables.

Of the variables analyzed, sex was the only one that showed significant differences in the negative Work–Life interaction (*p* = 0.017; K = 0.383). The women reported a higher mean (M = 1.26; SD = 0.52) compared to the men (M = 1.06; SD = 0.48).

As for Work–Life conflict, differences were observed according to the variables of being a paid carer (*p* = 0.01; K = 0.369), the number of children, and the age of the nursing professional. The people who had external support for childcare showed greater Life–Work conflict (M = 0.64; SD = 0.45) compared to those who did not (M = 0.44; SD = 0.44). Similarly, greater conflict was observed the greater the number of children (r = 0.125*; *p* = 0.029) and the age of the professional (r = 0.113; *p* = 0.048). 

On the other hand, in the positive Work–Life interaction, significant differences were found in two variables: religion (*p* = 0.045; K = 0.431) and being a paid caregiver (*p* = 0.017; K= 0.624). The people with religious beliefs (M = 1.50; SD = 0.65) and those who did not delegate the care of their children obtained higher scores (M = 1.47; SD = 0.64).

In the positive Life–Work interaction, significant differences were observed according to the variables of sex (*p* = 0.028; K = 0.393), having children (*p* = 0.005; K = 0.406), and the number of children (r = 0.150; *p* = 0.009). The women reported a higher positive interaction (M = 1.99; SD = 0.66) than men (M = 1.65; SD = 0.85). Likewise, the people with children showed a higher positive interaction (M = 2.03; SD = 0.67) compared to those without children (M = 1.83; SD = 0.71). Furthermore, a greater number of children correlated with greater Life–Work enrichment (r = 0.150; *p* = 0.009).

The results related to the work and COVID-19 variables are shown in Table 5.

Differences were only observed in the conflict subscale. Thus, greater Work–Life conflict was observed in those who worked shifts (M = 1.31; SD = 0.52; *p* = 0.001; K = 0.392), those who worked night shifts (M = 1.29; SD = 0.53; *p* = 0.045; K = 0.434), and those who perceived greater stress (r = 0.324; *p* = 0.001). On the other hand, those who worked in services with low exposure (M = 0.50; SD = 0.47; *p* = 0.036; K = 0.572) and with a greater perception of stress (r = 0.214; *p* = 0.001) showed greater Life–Work conflict.

Table 6 presents the four multiple linear regression models for the different Work–Life and Life–Work interactions, in both their positive and negative dimensions. When all of the variables were introduced, the most significant ones were identified in each model, explaining 14%, 7%, 5%, and 5% of the variability in the results.

After estimating the models, collinearity diagnostics were conducted to assess the independence among the predictor variables. All tolerance values were close to 1.0 and all VIF (Variance Inflation Factor) values were approximately 1.0, indicating that multicollinearity was not a concern. The detailed diagnostic statistics are presented in Table 6.

In particular, in the Work–Life conflict, a significant positive association was established with the perceived stress variable (β = 0.323; 95% CI [0.035, 0.069]; *p* < 0.001). Likewise, working rotating shifts increased this conflict by 0.186 points (β = 0.186; 95% CI [0.085, 0.309]; *p* < 0.001).

With regard to the negative Life–Work interaction, the perceived stress again showed a significant positive association (β = 0.213; 95% CI [0.014, 0.044]; *p* < 0.001). Furthermore, for each child, there was an increase of 0.144 points in conflict (β = 0.144; 95% CI [0.014, 0.106]; *p* = 0.011).

For the positive work–home interaction, the paid carer variable showed a significant negative association of -0.143 points compared to those who did not have this help (β = −0.143; 95% CI [−0.503, −0.062]; *p* = 0.012). On the other hand, having religious beliefs showed a significant positive association, with an increase of 0.136 with respect to those who did not (β = 0.136; 95% CI [0.032, 0.327]; *p* = 0.018).

Finally, in terms of the positive Life–Work interaction, being a woman was significantly and positively associated with a better interaction of 0.162 points (β = 0.162; 95% CI [0.104, 0.559]; *p* = 0.004), as was having children (β = 0.148; 95% CI [0.051, 0.364]; *p* = 0.009).

## 4. Discussion

This study provides a comprehensive overview of the Work–Life Balance experienced by nursing staff during a particularly complex phase of the COVID-19 pandemic, the sixth wave in Spain, marked by the spread of the Omicron variant. This variant, characterized by its high transmissibility, led to an exponential rise in cases between December 2021 and February 2022. Although it exhibited lower lethality compared to that of previous variants, its rapid contagion overwhelmed healthcare services, placing unprecedented pressure on the healthcare system. In this context, the study assessed the interactions between perceived stress, sociodemographic and occupational variables, and their impact on nurses’ perceptions of Work–Life Balance.

In addition, the study sought to determine whether having contracted COVID-19 had a significant effect on the four dimensions of Work–Family Interaction (positive work-to-family, positive family-to-work, negative work-to-family, and negative family-to-work). Contrary to expectations, no statistically significant differences were found between those who contracted the virus and those who did not contract it in any of the four dimensions evaluated. This absence of effect may be attributed to several factors. First, the milder clinical manifestations associated with Omicron, in contrast to earlier variants, may have lessened the emotional and physical impact of infection, thereby reducing its direct influence on both work and family experiences (Serrano-Cumplido et al., 2024). Second, the high prevalence of infections during the sixth wave may have led to a process of collective normalization or adaptation, thereby reducing individual perceptions of vulnerability and the additional stress associated with personal infection (Ortiz-de-Lejarazu et al., 2024). Considering that stress has been highlighted as a key factor influencing both conflict and synergy in work–life relationships, this reduction in stress likely explains the absence of significant differences between infected and non-infected individuals in the work–family dimensions analyzed.

Another relevant factor analyzed in this study was spirituality, understood from a broad and inclusive perspective that goes beyond traditional religious affiliation. The predominance of non-religious beliefs among participants reflects Spain’s ongoing sociocultural evolution and underscores the importance of incorporating a non-religious view of spirituality into healthcare provision, staff well-being policies, and future research. The literature supports the notion that workplace spirituality positively influences nurses’ well-being and the quality of care they provide, as it is associated with a stronger sense of belonging, motivation, and commitment. In this regard, spiritual leadership has been identified as a key factor in fostering these outcomes (Ribeiro et al., 2021). Moreover, integrating spirituality into organizational dynamics promotes a sense of community and imparts greater meaning to work, yielding benefits at both the individual and institutional levels (Pirkola et al., 2016). The inclusion of spirituality-related content in nursing education and practice is essential to strengthening spiritual care competence, a core component of holistic health approaches (Lewinson et al., 2015; Rykkje et al., 2022). Given that professionals with religious affiliation tend to demonstrate higher competence in this area, it is necessary to develop inclusive educational strategies that address spirituality from an open, non-exclusively religious perspective (W. Wang et al., 2024).

From a labor perspective, it was observed that more than half of the participants had less than five years of experience in their respective departments, suggesting a high turnover rate likely linked to the structural precariousness of the Spanish healthcare system. This dynamic aligns with the findings of Rodríguez-Madrid et al. (2023) and Jiménez-García et al. (2022), who identified a high frequency of organizational changes in healthcare centers during the pandemic (Jiménez-García et al., 2022; Rodríguez-Madrid et al., 2023). In particular, Jiménez-García et al. (2022) highlights that 26.4% of nursing professionals were reassigned due to organizational reasons or vulnerability-related circumstances. This employment instability was further exacerbated during the sixth wave of COVID-19, during which one-third of the sample experienced sick leave or isolation, significantly increasing both the care burden and stress levels among staff. It is important to note that 80% of the participants reported moderate stress levels; this may have intensified conflicts between professional and personal spheres. The low prevalence of severe stress should be interpreted with caution, as it may reflect a “valley” phenomenon or underreporting in a context marked by healthcare system overload.

In this high-demand professional context, family environment conditions also played a decisive role. The way in which professional and family responsibilities intersect became a critical factor influencing both the psychological well-being and professional performance of nursing staff.

Indeed, one of the main contributions of this study lies in its multidimensional approach to Work–Life Balance, which considers both the negative and positive interactions between these two spheres. This perspective broadens the understanding of the phenomenon beyond conflict, also integrating the mutual benefits that may arise from the simultaneous exercise of professional and family roles. The analysis reveals that positive interactions, particularly those in which personal life enriches work life, yielded the highest scores. This suggests that even in high-pressure contexts such as the sixth wave, family resources may act as buffers against occupational stress. While family can serve as a source of support, the literature has also documented the less favorable side of these responsibilities in crisis contexts. The study by Marsden et al. (2022), conducted in Tasmania (Australia), found that home and family stressors were strong predictors of psychological distress among nurses during the COVID-19 pandemic. This research emphasizes that the support received within the home environment and the quality of family relationships can play a crucial role in preserving healthcare workers’ mental health (Marsden et al., 2022). Similarly, Avery et al. (2021), in a study conducted in the United States, found that women with children at home reported significantly higher levels of anxiety during the early months of the pandemic compared to those without children. These findings demonstrate that the presence of additional family responsibilities can increase emotional vulnerability in crisis contexts.

Nonetheless, our findings offer a complementary perspective that highlights the complex and multifaceted nature of Work–Life dynamics. It is particularly noteworthy that women and individuals with children reported higher levels of positive interaction from personal life to work. This result suggests that, despite the overload caused by school closures and increased caregiving demands, the family environment in many cases served as a facilitator of professional performance. In line with this interpretation, Nelson et al. (2013) and, more recently, Gartzia (2024), identified an association between the experience of parenthood and the development of key competencies for nursing practice, such as empathy, emotional regulation, and the ability to provide containment (Gartzia, 2024; Nelson et al., 2013). This approach underscores the importance of recognizing parenthood as a relevant dimension of leadership, and the need to adapt organizational practices to support caregiving responsibilities, ensuring that motherhood (or fatherhood) is not treated as a professional penalty (Gartzia, 2024).

In contrast, negative interaction from personal life to work showed generally low scores, but was significantly higher among those with a greater number of children or who delegated their care. Notably, outsourcing caregiving tasks to paid caregivers, rather than reducing conflict, was associated with a marked increase in Work–Life conflict. While delegating family responsibilities might be expected to alleviate strain, this decision can give rise to new concerns related to the quality of care, the need for constant supervision, and feelings of inadequacy in the parental role (Hughes et al., 2023). This suggests that not only the quantity but also the way in which family responsibilities are managed influences Work–Life Balance and the emotional experience of healthcare professionals.

Similarly, it was found that nurses assigned to services with low exposure to COVID-19 experienced greater family-to-work conflict. This finding may appear counterintuitive, as studies such as that by Liu et al. (2022) associate work in high-risk contagion areas with increased conflict between family and professional roles. However, others, such as J. Wang et al. (2024), align with our results by reporting high levels of conflict in Primary Care settings, which, according to the risk classification proposed by González-Pando et al. (2022), are considered low-risk (González-Pando et al., 2022; J. Wang et al., 2024). This categorization was used as a reference in our analysis. One possible explanation for this apparent discrepancy is that assignment to lower-risk areas may have led families to perceive nurses as less overburdened and more available at home. This perception may have resulted in additional pressure to assume domestic responsibilities, thereby increasing family demands and exacerbating Work–Life conflict.

Perceived stress had a significant impact on the negative dimensions of Work–Life Balance, highlighting how emotional fatigue resulting from care overload diminished professionals’ ability to adequately respond to the demands of the family environment. This effect was particularly pronounced among those working rotating or night shifts, a common modality in the nursing profession that was especially intensified during the sixth wave, marked by the proliferation of extraordinary shifts and substitutions due to infections. The literature has consistently documented that rotating shifts are associated with higher levels of Work–Life conflict, primarily due to the difficulties they pose in balancing childcare and domestic responsibilities (Clendon & Walker, 2017; Fujimoto et al., 2008). Similarly, night work has been linked to increased levels of conflict within the family sphere, underscoring the critical role that working conditions play in achieving reconciliation between one’s professional and personal life (Al-Hammouri & Rababah, 2023).

From a gender perspective, the conflict between personal life and work was greater among the women, highlighting the structural inequalities that persist in the distribution of family responsibilities. This finding is consistent with previous studies indicating that women experience greater Work–Life conflict compared to men, reflecting the gendered impact of such situations (Hwang & Yu, 2021; Yao et al., 2024). The persistence of social expectations that place a greater domestic burden on women increases the pressure to balance both spheres, creating a “double burden” that is not equitably distributed and remains a major source of inequality in both work and family life (Cezar-Vaz et al., 2022; J. Cohen & Venter, 2020). This inequality was further exacerbated during the public health emergency, where the absence of adequate Work–Life Balance measures worsened the situation. All of this underscores the urgent need to implement more equitable and flexible public and organizational policies that specifically address gender-differentiated needs (Naegle et al., 2023).

The findings of this study acquire particular significance when contextualized within a critical phase of the pandemic. The sixth wave not only highlighted the resilience of nursing staff but also exposed the structural vulnerabilities of the Spanish healthcare system and the human limits of those who sustain it.

However, to date, the literature on stress management and Work–Life Balance in this crisis context remains scarce, especially regarding the sixth wave and its specific impact on work and family dynamics.

Today, far from being resolved, many of these tensions have worsened. Recent reports reflect growing distress among nurses, chronic fatigue, and a sense of institutional abandonment, which has led a significant number of professionals to consider leaving the profession. In this scenario, Work–Life Balance emerged as a key dimension for well-being, professional retention, and quality of care.

Nonetheless, this dimension has historically been overlooked in healthcare policies, despite nursing being a predominantly female profession subjected to highly demanding working conditions. It is urgent to recognize that Work–Life Balance is not a private matter but a structural axis that directly affects occupational health, professional motivation, and the quality of care. Ignoring this reality not only deepens professional disengagement but also jeopardizes the system’s capacity to respond to future crises. Such measures should not be considered exceptional concessions but rather strategic pillars to ensure talent retention, care quality, and the sustainability of the public health system.

Therefore, the results of this study should be understood as a call to action for institutional decision-makers. It is urgent to advance towards organizational policies that ensure more humane, predictable work environments compatible with personal life. Work–Life Balance can no longer be addressed as an individual responsibility but must be recognized as a structural axis that directly impacts occupational health, professional motivation, and quality of care.

Among the priority measures is the genuine flexibilization of shifts through strategies such as compressed workweeks, self-managed scheduling, and the possibility of adjusting working hours according to family needs. These options help reduce the conflict between personal and work life and have been shown to be key in improving healthcare staff well-being (Dhaini et al., 2018).

Simultaneously, guaranteeing access to childcare services is essential, whether by establishing centers within the workplace or through agreements with nearby facilities that offer priority access and affordable rates. These measures not only facilitate Work–Life Balance but also promote more inclusive and sensitive work environments responsive to staff needs (Dousin et al., 2021).

Furthermore, parental leave policies must be strengthened, surpassing the established legal minimums. It is crucial to ensure that both women and men have sufficient time to fulfill their family responsibilities without this hindering their professional development. Equity in these policies contributes not only to reducing Work–Life conflict but also to a fairer distribution of caregiving tasks (Xiao et al., 2023).

Alongside this, structured emotional well-being programs must be implemented to actively address the impact of occupational stress. Initiatives such as mindfulness, psychological support, and cognitive restructuring contribute to better emotional management and the prevention of chronic burnout (Aranda Auserón et al., 2018; Gómez del Pulgar & Meléndez Moreno, 2017). In this regard, it is also essential to promote the right to digital disconnection, ensuring that workers can fully disengage from their work obligations outside of working hours. This measure is particularly relevant in an environment where the boundaries between personal and professional life are increasingly blurred (Blake et al., 2024; Torres & Rojas-Solís, 2024; Trujillo Pons, 2021).

It is also crucial that professional trajectories do not penalize motherhood or caregiving responsibilities. To this end, more flexible career models should be promoted, including part-time leadership roles or job-sharing schemes in managerial positions, allowing the reconciliation of professional development with family life (Chacón-Henao et al., 2022).

Finally, special attention must be given to the most vulnerable groups, such as those with greater family responsibilities or those working rotating shifts, who are at higher risk of experiencing conflicts between their personal and work lives (J. Cohen & Venter, 2020; Gifkins et al., 2017). Caring for nursing staff is not only an ethical imperative but also an essential condition for providing quality care to society.

## 5. Limitations

This study has several limitations that should be considered when interpreting its results. Firstly, due to the geographical dispersion of the participants, it was decided to use self-administered questionnaires for data collection. Although this methodology allowed access to a more diverse and representative sample of the healthcare reality, particularly in a context of the decentralization of competences in Spain, it is relevant to mention that self-administered questionnaires can be subject to biases, such as social desirability, which could influence the veracity of the responses and, therefore, the accuracy of the data obtained.

Additionally, the snowball sampling method, although effective in accessing hard-to-reach populations, carries an inherent risk of selection bias. This is because the initial participants, recruited through a pre-existing network of contacts, may tend to select individuals with similar characteristics to their own. This could result in a sample that does not adequately reflect the diversity of the target population, affecting the generalization of the results. To mitigate this bias, several strategies were employed, such as sending standardized invitations to the 52 offices of the nursing associations and the national and provincial branches of the SATSE union. This allowed for broader dissemination of the survey across various regions and healthcare settings (hospital and non-hospital). However, a concentration of responses was observed in certain provinces, such as Ceuta, Granada, Lugo, Madrid, and Valencia, which may have introduced a regional bias. To reduce this selection bias, the variable “place of work” was dichotomized into “mainland” and “non-mainland”, allowing for a more balanced analysis between groups. However, this simplification limited the ability to detect more specific differences within regions, especially in the non-mainland areas.

Furthermore, the cross-sectional approach used in the research limits the possibility of follow-up over time, making it difficult to identify causal relationships and to evaluate changes in the interaction between work and life in nursing staff during the pandemic. Likewise, the lack of longitudinal studies addressing the different phases of the health crisis prevents a deeper understanding of the evolution of the Work–Life Balance of health professionals and complicates the identification of patterns of adaptation to changes in working and social conditions over time. These limitations highlight the need for future research that adopts a longitudinal approach to capture the complexity of the situation in contexts of health crisis and that addresses the logistical challenges associated with the Spanish healthcare reality, in order to provide a more complete and dynamic view of the phenomenon analyzed.

Additionally, the relatively low explanatory power of the multiple regression models (R^2^ values between 5% and 14%) indicates the probable existence of key variables that were not included in the present study. Although a wide range of sociodemographic, occupational, and contextual variables were considered, other relevant factors could play a significant role in understanding Work–Life Balance. Future research should aim to integrate these dimensions to develop more comprehensive models that better capture the complexity of the phenomenon.

## 6. Conclusions

This study represents a significant contribution to the field of Work–Life Balance in nursing professionals, particularly in the highly demanding context of the sixth wave of the COVID-19 pandemic in Spain. The findings indicate that moderate levels of perceived stress, while not extreme, have a substantial impact on the perception of imbalance between work and family life. This highlights the importance of addressing stress levels as a key mediating factor in work–life dynamics.

The results also underscore the relevance of positive interactions between work and family domains, which appear to have a greater influence on overall well-being than negative interactions. This emphasizes the importance of fostering conditions that promote mutual enrichment between professional and family roles.

Another noteworthy finding is the buffering role of the family environment, which served as a source of emotional support during extreme work conditions. Additionally, the emergence of spirituality, particularly in its secular form, as a relevant psychosocial resource is highlighted, an aspect rarely addressed in the existing literature. The study also reveals that the outsourcing of childcare, rather than alleviating the burden, may contribute to increased subjective distress. This calls into question common assumptions regarding the delegation of care as an effective work–life balance strategy.

From a practical perspective, labor policies that promote greater scheduling flexibility and provide specific childcare resources are essential to reducing Work–Life conflict, especially in a workforce subjected to long shifts and rotating schedules. The high prevalence of shift and night work, combined with persistent moderate stress levels, points to the need for more targeted interventions focused on managing occupational stress and improving working conditions.

From a public policy standpoint, the results highlight the urgent need to design and implement institutional work–life balance policies that go beyond individual or informal measures. These should include structural solutions which includes flexible shift arrangements, access to childcare services adapted to hospital systems, sustainable mental health programs and effective guarantees of the right to disconnect from work. Such measures must be embedded within an organizational framework of shared responsibility, as they are essential to safeguarding the mental health of healthcare workers and preventing professional disengagement and attrition from the healthcare system.

Finally, future research should focus, once the policies recommended based on our findings have been implemented, on the development of longitudinal studies to examine the evolution of these strategies and their cumulative effects on professional well-being. Additionally, further investigation of other potentially relevant variables is recommended, given the limited explanatory power observed in the current models, with the aim of developing more comprehensive models that better capture the complexity of the phenomenon.

## Figures and Tables

**Table 1:** Sociodemographic variables.

	Participants (*n* = 305)
	M (SD)
Age (years old)	38.8 (11.383)
	***n* (%)**
Gender	
Man	40 (13.1)
Woman	265 (86.8)
Population	
Peninsular	232 (76.1)
Overseas territories	73 (23.9)
Religion	
With religious beliefs	130 (42.6)
Without religious beliefs	175 (57.4)
Relationship status	
Living with a partner	228 (74.7)
Living without a partner	77 (25.2)
Children	
Yes	175 (57.4)
No	130 (42.6)
Paid Caregiver	
Yes	35 (11.5)
No	270 (88.5)
	**M (SD)**
Number of children	1.03 (1.062)

**Table 2:** Labor variables and COVID-19.

	Participants *n* (%)
Years in service	
<5 years	168 (55.1)
>5 years	137 (44.9)
Department in which participant works	
Low-exposure	203 (66.6)
High-exposure	102 (33.4)
Working day	
Full day	262 (85.9)
Half day	23 (7.5)
Other	20 (6.6)
Rotating shift	
Yes	182 (59.7)
No	123 (40.3)
Nocturnal	
Yes	161 (52.8)
No	144 (47.2)
Management	
Yes	49 (16.1)
No	252 (82.6)
COVID-19 diagnosis	
Yes	54 (17.7)
No	251 (82.3)
Have you had to self-isolate?	
Yes	103 (33.8)
No	202 (66.2)

**Table 3:** Work–Life Balance and perceived stress variables.

	M (SD)
Work–Life Balance	
Negative Work–Life Balance	1.24 (0.519)
Negative Life–Work Balance	0.46 (0.443)
Positive Work–Life Balance	1.44 (0.634)
Positive Life–Work Balance	1.95 (0.693)
Perceived Stress	23.2 (3.24)
	***n* (%)**
Perceived Stress Classification	
Low stress	69 (22.6)
Medium stress	234 (76.7)
High stress	2 (0.7)

**Table 4:** Work–Life Balance according to sociodemographic variables.

	Negative Work–Life Balance			Negative Life–Work Balance			Positive Work–Life Balance			Positive Life–Work Balance		
	M (SD)	*p*	K	M (SD)	*p*	K	M (SD)	*p*	K	M (SD)	*p*	K
Gender												
Man	1.06 (0.48)	0.017 *	0.383	0.38 (0.53)	0.058		1.48 (0.67)	0.539		1.65 (0.85)	0.028 *	0.393
Woman	1.26 (0.52)			0.48 (0.43)			1.43 (0.63)			1.99 (0.66)		
Population												
Peninsular	1.25 (0.50)	0.329		0.47 (0.42)	0.227		1.43 (0.2.64)	0.678		1.96 (0.68)	0.887	
Overseas territories	1.18 (0.58)			0.43 (0.50)			1.45 (0.61)			1.92 (0.73)		
Religion												
With religious beliefs	1.23 (0.53)	0.524		0.45 (0.46)	0.23		1.50 (0.65)	0.045 *	0.431	1.99 (0.70)	0.053	
Atheist/Agnostic	1.25 (0.49)			0.49 (0.41)			1.33 (0.59)			1.86 (0.68)		
Relationship status												
Lives with a partner	1.25 (0.52)	0.334		0.46 (0.43)	0.934		1.40 (0.63)	0.085		1.98 (0.70)	0.166	
Lives without a partner	1.19 (0.52)			0.47 (0.48)			1.54 (0.63)			1.86 (0.68)		
Children												
Yes	1.22 (0.54)	0.304		0.50 (0.47)	0.115		1.43 (0.64)	0.923		2.03 (0.67)	0.005 **	0.406
No	1.25 (0.49)			0.41 (0.41)			1.45 (0.63)			1.83 (0.71)		
Paid Caregiver												
Yes	1.36 (0.59)	0.348		0.64 (0.45)	0.01 **	0.369	1.18 (0.56)	0.017 *	0.624	1.86 (0.65)	0.349	
No	1.22 (0.51)			0.44 (0.44)			1.47 (0.64)			1.96 (0.70)		
	**r**	***p***		**r**	***p***		**r**	***p***		**r**	***p***	
Number of children	−0.075	0.194		0.125 *	0.029		0.001	0.98		0.150 **	0.009	
Age (years old)	−0.072	0.207		0.113 *	0.048		−0.033	0.57		0.092	0.109	

* *p* < 0.05; ** *p* < 0.005.

**Table 5:** Work–Life Balance according to labor variables and COVID-19.

	Negative Work–Life Balance			Negative Life–Work Balance			Positive Work–Life Balance			Positive Life–Work Balance		
	M (SD)	*p*	K	M (SD)	*p*	K	M (SD)	*p*	K	M (SD)	*p*	K
Years in service												
<5 years	1.22 (0.52)	0.627		0.43 (0.42)	0.131		1.50 (0.61)	0.078		1.95 (0.67)	0.75	
>5 years	1.25 (0.52)			0.51 (0.47)			1.36 (0.65)			1.94 (0.72)		
Service in which they work												
High exposure	1.28 (0.48)	0.085		0.38 (0.38)	0.036 *	0.572	1.52 (0.64)	0.079		1.95 (0.75)	0.589	
Low exposure	1.21 (0.54)			0.50 (0.47)			1.40 (0.63)			1.94 (0.66)		
Working day												
Full time	1.25 (0.52)	0.355		0.45 (0.44)	0.373		1.43 (0.62)	0.204		1.93 (0.69)	0.481	
Part time	1.26 (0.48)			0.54 (0.53)			1.36 (0.77)			1.90 (0.75)		
Other	1.07 (0.50)			0.55 (0.40)			1.67 (0.67)			2.16 (0.67)		
Rotating Shift												
Yes	1.31 (0.52)	0.001 **	0.392	0.47 (0.47)	0.997		1.45 (0.63)	0.604		1.96 (0.68)	0.666	
No	1.13 (0.51)			0.45 (0.40)			1.42 (0.64)			1.93 (0.71)		
Nocturnality												
Yes	1.29 (0.53)	0.045 *	0.434	0.48 (0.48)	0.963		1.46 (0.63)	0.323		1.96 (0.67)	0.682	
No	1.18 (0.51)			0.45 (0.40)			1.41 (0.64)			1.93 (0.72)		
Management												
Yes	1.19 (0.59)	0.27		0.41 (0.51)	0.162		1.47 (0.73)	0.888		1.95 (0.86)	0.57	
No	1.24 (0.51)			0.48 (0.43)			1.43 (0.62)			1.94 (0.66)		
COVID-19 diagnosis												
Yes	1.21 (0.56)	0.642		0.40 (0.39)	0.256		1.36 (0.63)	0.365		1.84 (0.70)	0.225	
No	1.24 (0.51)			0.48 (0.45)			1.45 (0.64)			1.97 (0.69)		
Have you had to self-isolate?												
Yes	1.30 (0.52)	0.203		0.53 (0.48)	0.076		1.47 (0.58)	0.278		1.89 (0.73)	0.294	
No	1.20 (0.51)			0.43 (0.42)			1.42 (0.66)			1.98 (0.68)		
	**r**	***p***		**r**	***p***		**r**	***p***		**r**	***p***	
Perceived stress	0.324 **	0.001		0.214 **	0.001		0.022	0.699		0.099	0.083	

* *p* < 0.05; ** *p* < 0.001.

**Table 6:** Multiple linear regression model for Work–Life Balance.

**Multiple Linear Regression Model for Negative Work–Life Balance**							
**Variable**	**β**	**Error Dev.**	**95% CI**		***p***	**Tolerance**	**VIF**
Perceived stress	0.323	0.009	0.035	0.069	<0.001	1.000	1.000
Rotating shift	0.186	0.057	0.085	0.309	<0.001	1.000	1.000
Durbin–Watson Test = 1.779; F = 23.678; *p* < 0.001							
**Multiple Linear Regression Model for Negative Life–Work Balance**							
**Variable**	**β**	**Error Dev.**	**95% CI**		***p***	**Tolerance**	**VIF**
Perceived stress	0.213	0.008	0.014	0.044	<0.001	1.000	1.000
Number of children	0.144	0.023	0.014	0.106	0.011	1.000	1.000
Durbin–Watson Test = 1.905; F = 10.647; *p* = 0.011							
**Multiple Linear Regression Model for Positive Work–Life Balance**							
**Variable**	**β**	**Error Dev.**	**95% CI**		***p***	**Tolerance**	**VIF**
Paid caregiver	−0.143	0.112	−0.503	−0.062	0.012	0.999	1.001
Religious beliefs	0.136	0.075	0.032	0.327	0.018	0.993	1.007
Years of service <5 years	0.118	0.072	0.008	0.293	0.038	0.993	1.007
Durbin–Watson Test = 1.82; F = 5.302; *p* = 0.001							
**Multiple Linear Regression Model for Positive Life–Work Balance**							
**Variable**	**β**	**Error Dev.**	**95% CI**		***p***	**Tolerance**	**VIF**
Woman	0.162	0.116	0.104	0.559	0.004	1.000	1.000
Having children	0.148	0.08	0.051	0.364	0.009	1.000	1.000
Durbin–Watson Test = 1.784; F = 7.697; *p* < 0.001							

## Data Availability

The data presented in this study are available on request from the corresponding author due to privacy.
